# Epidemiological Survey and Confirmation of Autochthonous Cases of Bovine Fasciolosis in the Serrana Mesoregion of Santa Catarina, Brazil

**DOI:** 10.3389/fvets.2022.933462

**Published:** 2022-07-22

**Authors:** Larissa Américo, Mayckon Antonio Cardoso Padilha, Paula Maciel Arruda, Guilherme Drescher, Anderson Barbosa de Moura, Andreas Lazaros Chryssafidis

**Affiliations:** ^1^Laboratory of Parasitology and Parasitic Diseases (LAPAR-CAV-UDESC), Department of Veterinary Medicine, Agroveterinary Sciences Center, Santa Catarina State University, Lages, Brazil; ^2^Laboratory of Cellular Biology, Carlos Chagas Institute, Oswaldo Cruz Foundation (ICC-FIOCRUZ), Curitiba, Brazil

**Keywords:** *Fasciola hepatica*, prevalence, ruminants, zoonosis, one health

## Abstract

*Fasciola hepatica* is reported as a trematode of great importance, causing fasciolosis, one of the most neglected zoonotic parasitic diseases in the world. In Brazil, potential losses of around US$ 210 million per year were calculated, with bovine fasciolosis alone. The State of Santa Catarina is located in the South Region of Brazil, with a herd of more than 4 million ruminants. The Serrana Mesoregion, historically free of fasciolosis, is composed of 30 municipalities, which have a consolidated agricultural tradition, mainly in extensive ruminant livestock. The objective of the present work was to investigate the occurrence and distribution of autochthonous cases of bovine fasciolosis in the region. For this, different analyzes were carried out in rural properties from eight municipalities, and in three slaughterhouses under the State Inspection Service (SIE), which received animals from all the Serrana Mesoregion. The rural properties were randomly selected, and up to 20 fecal samples were collected from random cattle older than 1 year, for analysis by coproparasitological sedimentation test. The traceability records of the positives were checked, for identifying autochthonous cases. Additionally, the tracking data of cattle slaughtered between the years of 2018 and 2021, which presented liver condemnations, were analyzed for defining autochthonous cases of bovine fasciolosis. In total, there were fecal samplings in 106 farms, with 1,927 tested cattle. From these, 178 (9.2%) were positive and autochthonous, but the overall result did not reflect the heterogeneity found between the evaluated municipalities. During the analyzed period, 11,556 cattle were slaughtered, with 1,744 (15.1%) liver condemnations due to fasciolosis in animals that never left the Serrana Mesoregion. The present study confirmed the presence of autochthonous cases of bovine fasciolosis in municipalities of the Serrana Mesoregion, and verified a high rate of positivity in animals slaughtered in the regional slaughterhouses. When the prevalence of bovine fasciolosis *in vivo* was compared with the *postmortem* positivity index in five municipalities, the results disagreed in four municipalities (*p*-value < 0.05), emphasizing the need for field investigation for the better understanding of the distribution and frequency of the parasitosis.

## Introduction

Due to the great impact generated on human and animal health, especially in cattle, *Fasciola hepatica* is reported as a trematode of great importance, causing fasciolosis, one of the most neglected zoonotic parasitic diseases in the world (1–3). It is also integrating the list of neglected tropical diseases of the World Health Organization (WHO), in the group of trematodes caused by foodborne diseases (3).

The young parasite migrates through the liver parenchyma and the adult is located in the bile ducts of warm-blooded animals, such as sheep, goats, cattle, buffalo, swine and humans (4, 5). *F. hepatica* has an indirect life cycle, involving freshwater snails of the Lymnaeidae family as intermediate hosts (4).

An annual economic impact of more than US$3.2 billion on world ruminant production is estimated from liver condemnation due to fasciolosis (6). In Brazil, a loss of around US$ 210 million per year was calculated with bovine fasciolosis alone (7). Among the States with the highest losses, related to the decrease in carcass weight and liver condemnations, there are Rio Grande do Sul, Espírito Santo and Santa Catarina, in decreasing order (7).

The State of Santa Catarina is located in the South Region of Brazil, and has a herd of more than 4 million ruminants (8). Fasciolosis is endemic and presents a high incidence in the coastal strip of the State (9–11), mainly due to climatic factors that influence the development of gastropod vectors (9, 12, 13).

The Serrana Mesoregion, located at the center-south of Santa Catarina State, is composed of 30 municipalities (14) that have a consolidated agricultural tradition, mainly in extensive ruminant livestock (15). There are studies that reported liver condemnation due to fasciolosis in cattle slaughtered in this region, in slaughterhouses under Federal Inspection Service (9–11, 16). However, these slaughterhouses receive cattle from many different regions, eventually from the Rio Grande do Sul State, and there was no investigation about the origin or movement of such animals, thus there was no confirmation of autochthonous cases.

Given the importance of fasciolosis for the economy, animal and human health, it is essential to assess the occurrence of the disease in the animal population and define the parasite distribution, providing support for the design of control strategies. Thus, the objective of the present work was to investigate the occurrence and distribution of autochthonous cases of bovine fasciolosis in the Serrana Mesoregion, as well as to compare the *in vivo* examination with the *postmortem* analysis of bovines for the diagnostic of fasciolosis.

## Materials and Methods

The protocols used in this investigation were approved by the Ethics Committee for the Use of Animals of the Agroveterinary Sciences Center, Santa Catarina State University (protocol CEUA N° 5381070619).

### General Local Characterization

The Serrana Mesoregion is composed of 30 municipalities (14). The Serrano climate is classified according to Köppen-Gelger as Temperate Oceanic Climate, that is, temperate and constantly humid (17), and the plant typology is classified as mixed ombrophilous forest, also called araucaria forest (18, 19). The municipalities included in the *in vivo* sampling were selected according to their geographic location and access limitations imposed by the pandemics of COVID-19. Thus, fecal samplings were carried out in the municipalities of Anita Garibaldi, Campo Belo do Sul, Capão Alto, Bocaina do Sul, Lages, Painel, São Joaquim and Urupema. For the *post mortem* analysis, the traceability records of cattle slaughtered from the 30 municipalities were checked.

### Fecal Sampling of Bovines

For the fecal samplings and diagnosis of *F. hepatica* infection, cattle from eight municipalities of the Serrana Mesoregion were sampled between August 2019 and November 2021. Up to 20 animals aged over 12 months were randomly selected from random farms included in the study. The farms were selected from different regions within the municipalities, with the aid of technicians from Agricultural Research and Rural Extension Company of Santa Catarina (EPAGRI).

For the sample size calculation, the total number of ruminants and producers per municipality in the Serrana Mesoregion was kindly provided by the Integrated Agricultural Development Company of Santa Catarina (CIDASC). The sample size of tested cattle per municipality was calculated according to the prevalence estimate by simple random sampling (20), with the expected prevalence of 4.5% of bovine fasciolosis in Santa Catarina (9), and error of 5%, according to the formula, where *n* is the sample size for an infinite population, *P*_*exp*_ is the expected prevalence and *d* is the error:


(1) $$\begin{array}{l} n=\frac{{1,96}^{2}P_{\exp}\left(1-P_{\exp}\right)}{d^{2}} \end{array}$$

Subsequently, the correction for finite populations was applied to obtain the adjusted sample size, according to the following formula, where *n*_*adj*_ is the adjusted sample, *N* is the total population and *n* is the sample size for an infinite population:


(2) $$\begin{array}{l} n_{adj}=\frac{N*n}{N+n} \end{array}$$

The fecal samples were collected individually, directly from the rectal ampulla, with the aid of long palpation gloves. After collection, the samples were stored in a thermal box and transported to the Laboratory of Parasitology and Parasitic Diseases, Agroveterinary Sciences Center, Santa Catarina State University, where they were stored at 4°C until their processing, which occurred within 72 h at maximum.

### Coproparasitological Analysis

For the *in vivo* diagnostic of bovine fasciolosis, an adapted protocol of coproparasitological analysis by sedimentation was used, with double of the sample quantity used in the original protocol, and reading the entire sediment under the stereomicroscope, for improving the sensitivity of the test (21–23).

Briefly, for the coproparasitological sedimentation test, 6 g of feces was homogenized in 84 mL of tap water, inside a 250 mL graduated polyethylene column, using a glass rod. The content was filtered through a tea strainer and placed in a 500 mL conical glass flask. Then, the volume was adjusted to 400 ml with tap water, and the sample was allowed to settle for 10 min. The supernatant was discarded by careful eversion, leaving approximately 50 ml with the sediment in the conical flask, and the procedure was repeated twice, to clean up the sample. After the last eversion, the remnant sample was left to settle for approximately 5 min and the supernatant was carefully removed with a disposable Pasteur pipette. For reading, the whole sediment was distributed in two Petri dishes, each one stained with three drops of 1% Methylene Blue. The entire sample was read under a stereomicroscope (Stremi 508, Zeiss) and, whenever necessary, the parasite structures were collected with a micropipette, transferred to a slide and the morphological confirmation of *F. hepatica* eggs was carried out under a microscope (E200, Nikon).

### Analysis of Cattle Slaughtered in the Serrana Mesoregion, Under the State Inspection Service (SIE)

For the analysis of liver condemnations in slaughterhouses located in the Serrana Mesoregion, the CIDASC kindly provided a report of cattle slaughtered in the region between June 2018 and July 2021. The report contained the total number of slaughtered cattle in the selected abattoirs, the inspection notes, such as liver condemnations because of fasciolosis, the individual tracking tag number of every animal, and all their movement between farms or municipalities, according to the Brazilian System of Beef and Buffalo Meat Origin Identification and Certification (SISBOV) and Management System for Agricultural Defense of Santa Catarina (SIGEN+).

The analyzed data came from three slaughterhouses under the State Inspection Service (SIE) located in the municipalities of Lages, Otacílio Costa and São Joaquim. These slaughterhouses receive animals from the municipalities of the Serrana, Sul, Oeste and Vale do Itajaí Mesoregions. In the study, data from the municipalities of: Abdon Batista, Anita Garibaldi, Bocaina do Sul, Bom Jardim da Serra, Bom Retiro, Brunópolis, Campo Belo do Sul, Campos Novos, Capão Alto, Cerro Negro, Correia Pinto, Curitibanos, Frei Rogério, Lages, Otacílio Costa, Painel, Palmeira, Ponte Alta, Ponte Alta do Norte, Rio Rufino, Santa Cecília, São Cristovão do Sul, São Joaquim, São José do Cerrito, Urubici, Urupema, Vargem and Zórtea. All these municipalities are located in the Serrana Mesoregion.

### Traceability and Confirmation of Autochthonous Cases of Bovine Fasciolosis

To confirm autochthonous cases of bovine fasciolosis in the Serrana Mesoregion, all animals sampled in the field and animals that had liver condemned for fasciolosis had their individual earring identification number (SISBOV) analyzed in SIGEN+ system. In this system, the complete life history of the animal was investigated, from the birth to the moment of fecal sampling or slaughtering, to assess the origin and movement of the animals. Autochthonous cases were defined when the infected bovine did not transit in any municipality outside the Serrana Mesoregion.

### Frequencies of Bovine Fasciolosis

All data were stored and organized in Excel 2013 spreadsheets (Microsoft) for further analysis and tables preparation. The prevalence (P) of bovine fasciolosis was calculated with the data from the field random sampling, while the bovine liver condemnations due to fasciolosis were used to calculate the positivity index (PI). The postmortem data was not used to calculate prevalence, as we considered that the cattle sent for slaughtering would not represent the entire population of their municipalities of origin. Both P and PI were calculated according to the number of positives, being infected animals (IA) or condemned livers (CL), respectively, in relation to the total number of sampled animals (TSA) or total number of slaughtered animals (TNA) from the same municipality, according to the following formula:


(3) $$\begin{array}{l} P/IP=\frac{IA/CL}{TSA/TNA}\times 100 \end{array}$$

### Comparison Between *in vivo* and Post Mortem Analysis

In the municipalities of Bocaina do Sul, Capão Alto, Lages, Painel and Urupema, which presented positive animals in the *in vivo* and *post mortem* analysis, *Pearson's* chi-square (*X*^2^) test and *Fisher's* exact test were applied to verify the agreement between the two approaches for investigating the frequency of *F. hepatica* in populations of cattle. All analyzes were performed using the R statistical software [version 3.6.3, (24)], with a significance level of 5%.

### Assessment of Potential Economic Impact

To determine the potential economic losses generated by bovine fasciolosis in the Serrana Mesoregion, the overall prevalence found in the study was extrapolated to the total herd of the analyzed region (8), and the impact calculated based on US$56.6 per animal positive for *F. hepatica* (7).

### Geolocation and Distribution of Bovine Fasciolosis in the Serrana Mesoregion

Representative maps of the prevalence and positivity index of each municipality were produced with the software QGIS 3.20 (Geographic Information System), and the results were subdivided into groups of low (0.1–5.0%), medium (5.1–10 0.0%) and high (>10.1%) positivity (25).

## Results

### Analysis of Bovine Data *in vivo* and *post mortem*

A total of 106 livestock properties in eight municipalities in the Serrana Mesoregion were visited. The sample size for the infinite population was *n* = 66.04, given the regional prevalence of bovine fasciolosis of 4.5%, described in previous literature (9). The adjusted *n* according to the population was the same, and it is described in Table 1. The minimum sampling was extrapolated in most of the municipalities included in this project (Table 1).

**Table 1:** Sample calculation based on the bovine population in eight municipalities in the Serrana Mesoregion.

**Counties**	**Bovine population of the serrana mesoregion**	***n-adjusted***	***n-collected***
Anita Garibaldi	40.100	66	216
Bocaina do Sul	21.324	66	34^*^
Campo Belo do Sul	35.966	66	20^*^
Capão Alto	40.325	66	284
Lages	112.895	66	466
Painel	44.655	66	423
São Joaquim	90.251	66	90
Urupema	23.781	66	394

*n, sample number*;

* *The minimum sample number was not reached*.

**Figure 1:**
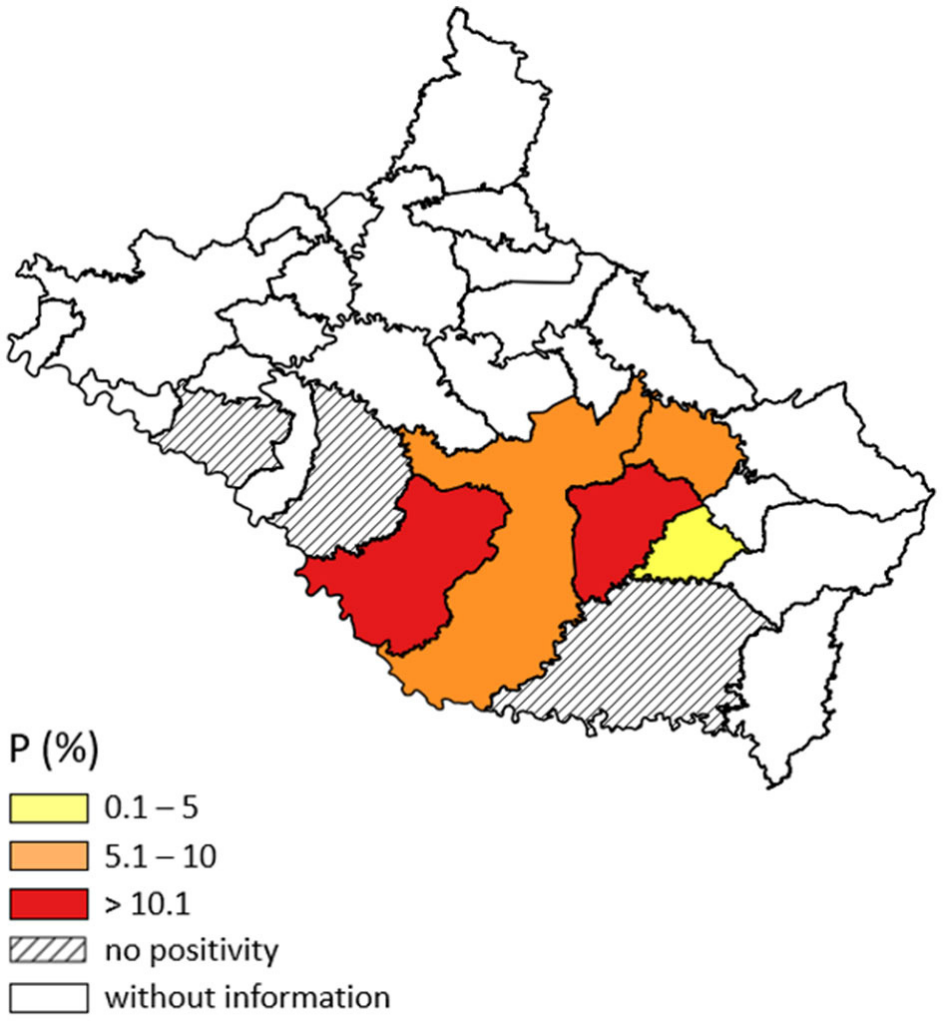
Map of the distribution of bovine fasciolosis in the eight municipalities of the Serrana Mesoregion of Santa Catarina (SC) evaluated in the present study. Prevalence found in cattle analyzed in the field, in random properties, from 2019 to 2021.

In total, 1,927 cattle were tested by coproparasitological sedimentation examination, of which 178 were autochthonous, with an overall prevalence of 9.2% (178/1.927). The municipalities with high prevalence (>10.1%) were: Painel (24.3%; 103/423) and Capão Alto (11.2%; 32/284); and average prevalence: Lages (7.51%; 35/466) and Bocaina do Sul (5.88% 2/34) (Table 2 and Figure 1).

**Table 2:** Prevalence of *Fasciola hepatica* through egg research in fecal samples of naturally infected cattle in municipalities in the Serrana Mesoregion, between 2019 and 2021.

**Counties**	**Sampled animals**	**Positive animals**	**Prevalence (%)**
Anita Garibaldi	216	0	0, 0
Bocaina do Sul	34^*^	2	5, 8
Campo Belo do Sul	20^*^	0	0, 0
Capão Alto	284	32	11, 2
Lages	466	35	7, 5
Painel	423	103	24, 3
São Joaquim	90	0	0
Urupema	394	6	1, 5
Total	1.927	178	9, 2

* *The minimum sample number was not reached*.

Another point to be highlighted is the type of cattle breeding in the region, being observed that most producers raise their animals extensively, with the main sources of water for the animals in rivers, streams, springs and dams.

Based on data from the three slaughterhouses under SIE, located in the Serrana Mesoregion, 11,556 cattle were slaughtered between June 2018 and July 2021, with 1,744 animals with condemned livers due to the presence of the parasite and lesions generated by fasciolosis. Of the 28 municipalities included in the study, 23 of them were positive (Table 3). Only animals that transited in the municipalities of the Serrana Mesoregion were counted, thus characterizing all positive animals as autochthonous.

**Table 3:** *Fasciola hepatica* positivity index in cattle in the slaughter line of three slaughterhouses of the State inspection system in municipalities in the Serrana Mesoregion.

**Counties**	**Sampled animals**	**Positive animals**	**Positivity index (%)**
Abdon Batista	4	0	0
Anita Garibaldi	46	1	2, 1
Bocaina do Sul	61	40	65, 5
Bom Jardim da Serra	137	3	2, 1
Bom Retiro	134	3	2, 2
Brunópolis	309	11	3, 5
Campo Belo do Sul	101	3	2, 9
Campos Novos	121	9	7, 4
Capão Alto	641	93	14, 5
Cerro Negro	27	6	22, 2
Correia Pinto	1.088	188	17, 2
Curitibanos	953	60	6, 2
Frei Rogério	59	0	0
Lages	1.917	357	18, 6
Otacílio Costa	956	181	18, 9
Painel	325	133	40, 9
Palmeira	693	194	27, 9
Ponte Alta	569	182	31, 9
Ponte Alta do Norte	21	0	0
Rio Rufino	8	0	0
Santa Cecília	23	17	73, 9
São Cristovão do Sul	169	17	10
São Joaquim	1.632	67	4, 1
São José do Cerrito	1.379	164	11, 8
Urubici	100	6	6
Urupema	12	4	33, 3
Vargem	70	5	7, 1
Zórtea	1	0	0
Total	11.556	1.744	15, 1

**Figure 2:**
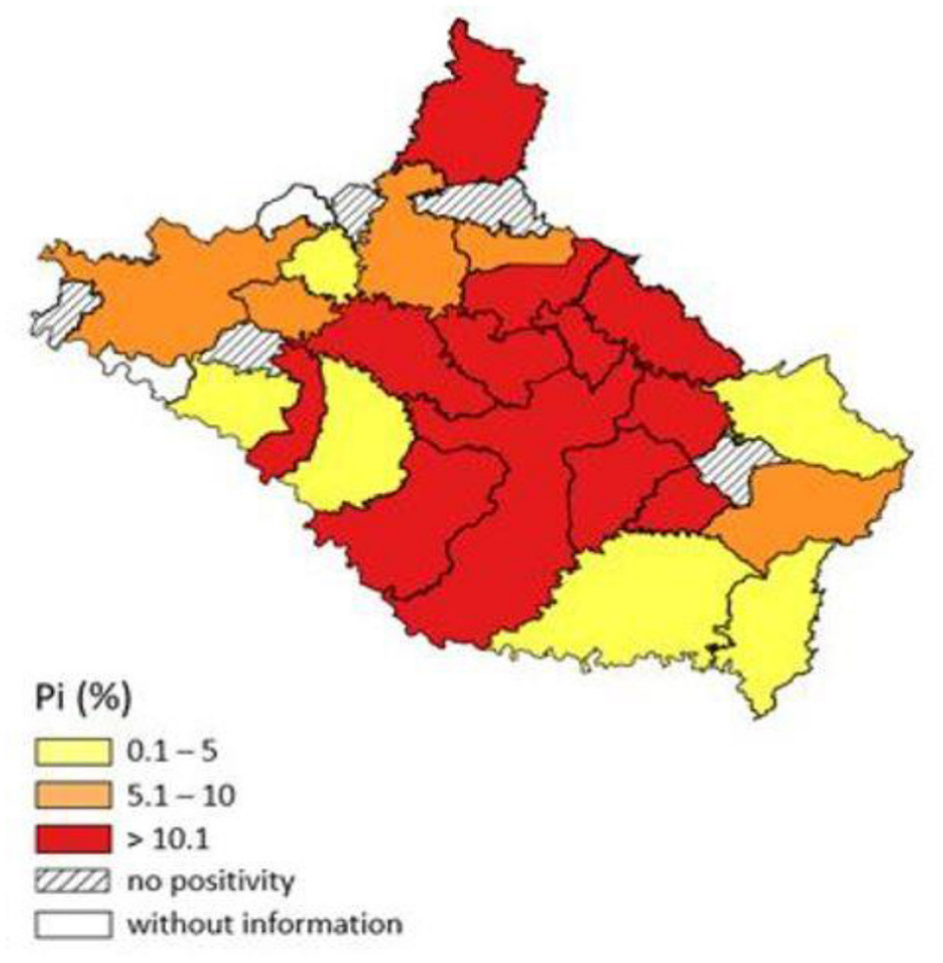
Map of the distribution of the bovine fasciolosis positivity index from 2018 to 2021 according to data from three slaughterhouses under the State Inspection Service in 28 municipalities in the Serrana Mesoregion of Santa Catarina.

In the analysis of data from slaughterhouses, it was found that the general PI of the 23 municipalities with infected animals was 15.1% (1.744/11.556) in the Serrana region. The municipalities that showed a high rate of positivity (>10.1%) (in red, Figure 2) were Santa Cecília (73.9% 17/23), Bocaina do Sul (65.5% 40/61), Painel (40.9% 133/325), Urupema (33.3% 4/12), Ponte Alta (31.9% 182/569), Palmeira (27.9% 194/693), Cerro Negro (22.2% 6/ 27), Otacílio Costa (18.9% 181/956), Lages (18.6% 357/1.917), Correia Pinto (17.2% 188/1.088), Capão Alto (14.5% 93/641) and São José do Cerrito (11.8% 164/1.379). Only the municipalities of Abdon Batista, Frei Rogério, Ponte Alta do Norte, Rio Rufino and Zórtea did not present positive liver condemnation for fasciolosis.

### Concordance Analysis

In the study, the agreement between liver condemnation data and field sampling of positive and negative animals for *F. hepatica* in the municipalities of Bocaina do Sul, Capão Alto, Lages, Painel and Urupema was analyzed, using the chi-square test and the Fisher's exact test (Table 4).

**Table 4:** Comparison between the prevalence of bovine fasciolosis *in vivo* with the post mortem positivity index through chi-square and Fisher's exact test in the municipalities of Bocaina do Sul, Capão Alto, Lages, Painel and Urupema.

**Counties**	***p-*value chi-square**	***p-*value *Fisher***
Bocaina do Sul	6,65e−08^*^	5,21e−06^*^
Capão Alto	0,2203	0,211
Lages	9,82e−06^*^	6,05e−07^*^
Painel	1,98e−06^*^	1,76e−03^*^
Urupema	1,38e−06^*^	8,46e−02^*^
General	<2,2e−16^*^	<2,2e−16^*^

* *Significant difference*.

As a result, a significant difference was found (*p*-value < 0.05) with the results of the municipalities of Bocaina do Sul, Lages, Painel and Urupema. In Capão Alto, the *p*-value was not significant (chi-square *p* = 0.22; Fisher's *p* = 0.21) indicating agreement between the compared analyses. The results from the field samples of Anita Garibaldi, Campo Belo do Sul and São Joaquim could not be compared to the data from abattoirs using chi-square and Fisher's exact tests, as they were negative in the first analysis.

### Economic Losses

Fasciolosis greatly impairs bovine production, decreasing the weight gain, the final carcass weight and causing liver condemnation. The overall prevalence of bovine fasciolosis investigated and calculated in this study is of 9.2%. Extrapolating this prevalence to the Serrana Mesoregion, considering the regional bovine herd of 765,529 animals (8), and that fasciolosis generates a loss of US$ 56.6 dollars per infected bovine (7), a total loss of approximately U$ 4 million/year (U$ 3,986,281.40/year) can be estimated for the Serrana Mesoregion.

## Discussion

The Serrana Mesoregion of Santa Catarina State, Brazil, is a region with a very consolidated tradition of extensive ruminant farming, historically free of fasciolosis (26). In the present study, a very large number of animals were surveyed for bovine fasciolosis, by *in vivo* investigation with coproparasitological tests, and by the *post mortem* data analysis in slaughterhouses under the SIE.

In the present study, the average prevalence of positive infected animals through coproparasitological examination in eight municipalities in the Serrana Mesoregion was 9.2% (178/1,927), a high prevalence for a region previously considered free of fasciolosis. The number of tested animals in the municipalities of Bocaina do Sul and Campo Belo do Sul did not reach the minimum sampling required, but it occurred because of the COVID-19 pandemics and its restrictions. The general prevalence of the Serrana Mesoregion does not reflect the heterogeneity in the levels of fasciolosis found among the investigated municipalities.

Even though bovine fasciolosis does not commonly cause mortality in parasitized animals, it is still a highly important disease (1). Attention to infected animals is directly related to the great economic impact on production animals due to the decrease in milk production, reduction in weight gain and low fertility (16).

In the State of Santa Catarina, there were two studies with similar methodology, where live animals were analyzed by coproparasitological sedimentation examination. The first was a retrospective study with samples of cattle, buffaloes, goats and sheep sent to the laboratory of the Ministry of Agriculture, Livestock and Supply, over a period of 12 years. At that time, no sample from three municipalities of the Serrana Mesoregion (Curitibanos, Campos Novos and São Joaquim) and was positive, but an overall prevalence of 27.8% (1.994/7.156) infected cattle was found in Santa Catarina, especially along the coastal region (10). However, it seems that these stool samples were from animals presenting some clinical sign, which justifies the high rate of positivity.

The second study evaluated the prevalence of *F. hepatica* in cattle from five municipalities located in the extreme South of Santa Catarina, which are Turvo, Timbé do Sul, Meleiro, Jacinto Machado and Morro Grande. A total of 290 bovine stool samples were collected in 61 properties, and a prevalence of 30.4% (88/290) was indicated in that region (27).

None of these previous studies carried out a proper investigation of animals located in the Serrana Mesoregion, emphasizing the scarcity of data and information about the fasciolosis in this location. The present study had a specific focus on the Serrana Mesoregion, prioritizing the randomness of the animals collected, for a better evaluation of the prevalence of the disease, and the investigation of the complete animal transit, from their birth until the moment of collection. This protocol proved that the bovines are becoming infected in the specific region, highlighting the importance of the obtained results for the animal health and local economy.

The adapted coproparasitological examination of sedimentation (21) was chosen because of its specificity for the identification of the dense eggs of *F. hepatica*. To increase the sensitivity of the technique, twice the recommended stool volume was used, as recommended in the literature (23). Furthermore, for the reading of the samples, drops of Methylene Blue 1% were added to the samples, and the reading of the entire sediment in Petri dishes was carried out. This technique was used to facilitate the visualization of the eggs in relation to the color of the bottom of the plate, in which the sediment acquired a blue color and contrasted with the eggs that maintained the original yellowish color, thus increasing the precision of the laboratory diagnosis (28).

Through the coproparasitological examination of the animals *in vivo*, the study also contributed directly to the epidemiological control of the parasitosis through the diagnosis and treatment of the animals before slaughter, increasing the profitability of the local producers.

The Serrana Mesoregion is historically known for its large-scale farms dedicated to livestock (29). The cattle raised in the region is kept mainly in extensive grazing, being one of the main economic activities in the region for more than 150 years (30). This type of cattle management allows the animals to have direct contact with natural water sources, such as rivers, streams, dams and swamps. These type of environments favor the proliferation of gastropods, therefore, they significantly increase the chances of cattle infection by *F. hepatica* (31). Additionally, the temperate and humid climate of the Serrana Mesoregion is ideal for the development of vector gastropods (17).

In order to expand information on fasciolosis in the Serrana Catarinense Meserregion, data from SIE slaughterhouses located in the region were compiled in search of new data on liver condemnation by *F. hepatica*. Some studies have already been carried out previously referring to data from the Federal Inspection System (7, 9, 11, 26).

The Serrana Mesoregion of Santa Catarina, Brazil, was historically free of this parasite (26). Silva (11) considered that the entire State of Santa Catarina was endemic, based on data from the Federal Inspection System in regional slaughterhouses. However, there was no analysis of the movement of cattle, or any evidence of autochthony of positive bovines. The abattoirs receive animals from several different locations, and it is not possible to identify the origin of infection without investigating the transit of the infected animals.

The information from the present study demonstrated a relevant positivity index (PI) of fasciolosis in the studied region, much higher than the data previously described in the literature (11). According to SIE data, the estimated general PI was 15.1% (1.744/11.556) of infected animals located in 28 municipalities in the Serrana Mesoregion. Data from the Federal Inspection System for the years 2004 to 2008 and 2010 estimated the PI of 8.8% (24.455/518.635) of livers infected with *F. hepatica* throughout the State of Santa Catarina (11). The results of this previous study corroborate the present study only in the cities of Palmeira, Bocaina do Sul and Ponte Alta, which presented high PI in both studies.

The prevalence of bovine fasciolosis in Anita Garibaldi and São Joaquim was so low that no infected animal was detected by fecal examination, even with a random sampling above the minimum desired sample (Table 2). However, some positives were detected in the records of liver condemnation from the region. This comparison also emphasizes the necessity of field studies in order to investigate the correct distribution of the parasitosis in a determined region, as the *postmortem* data did not match the detected prevalence.

When comparing the results found in the field study (*in vivo*) with the results from the slaughterhouses (*postmortem*), a significant difference was detected between these approaches in the municipalities of Bocaina do Sul, Lages, Painel and Urupema, that is, there was no agreement between the *in vivo* and the *postmortem* analysis (Table 4). This lack of agreement highlights the importance of field research in search of the real epidemiological situation of animals that are in the production process.

## Conclusion

The present study confirmed autochthonous cases of *F. hepatica* infection in cattle in the Serrana Mesoregion, combining *in vivo* tests with *postmortem* investigation. The prevalence of bovine fasciolosis was evaluated in the region through random sampling of bovines, even extrapolating the minimum sample size, providing robustness to the obtained results. A high positivity index was verified in animals slaughtered in abattoirs under the State Inspection Service (SIE). However, the positivity index verified by liver condemnation and the real prevalence of fasciolosis in animals in the field were different, emphasizing the need for the field investigation in order to determine the real prevalence of bovine fasciolosis in a population. The detection of infected animals in a historically free region is a very important information, not only for the region economy and health, but for the understanding and control of *F. hepatica* distribution. More investigations are being carried out, for the identification of the activities and epidemiological factors that contributed for the occurrence of fasciolosis in the region. They will contribute for the planning and implementation of efficient measures to control this disease, thus reducing economic losses for cattle farmers, and protecting the human health by the prevention of possible zoonotic cases.

## Data Availability Statement

The raw data supporting the conclusions of this article will be made available by the authors, without undue reservation.

## Ethics Statement

The animal study was reviewed and approved by Ethics Committee for the use of animals of the Agroveterinary Sciences Center, Santa Catarina State University (protocol CEUA N° 5381070619). Written informed consent was obtained from the owners for the participation of their animals in this study.

## Author Contributions

AC designed the study. LA, PA, and AC carried out the field sampling and coproparasitological tests. LA, MP, and GD analyzed the post mortem data. LA, AM, and AC wrote the manuscript. All authors contributed to the article and approved the submitted version.

## Funding

The authors would like to thank FAPESC (grant number 027/2020−793/2021) for funding the materials used in the study. We would also like to thank CAPES for funding LA masters degree.

## Conflict of Interest

The authors declare that the research was conducted in the absence of any commercial or financial relationships that could be construed as a potential conflict of interest.

## Publisher's Note

All claims expressed in this article are solely those of the authors and do not necessarily represent those of their affiliated organizations, or those of the publisher, the editors and the reviewers. Any product that may be evaluated in this article, or claim that may be made by its manufacturer, is not guaranteed or endorsed by the publisher.
